# Development and validation of a screening model for diabetes mellitus in patients with periodontitis in dental settings

**DOI:** 10.1007/s00784-020-03281-w

**Published:** 2020-06-15

**Authors:** Naichuan Su, Wijnand J. Teeuw, Bruno G. Loos, Madeline X. F. Kosho, Geert J. M. G. van der Heijden

**Affiliations:** 1 Department of Social Dentistry, Academic Centre for Dentistry Amsterdam (ACTA), University of Amsterdam and Vrije Universiteit Amsterdam, Amsterdam, the Netherlands; 2 Department of Periodontology, Academic Centre for Dentistry Amsterdam (ACTA), University of Amsterdam and Vrije Universiteit Amsterdam, Amsterdam, the Netherlands

**Keywords:** Periodontitis, Diabetes mellitus, Screening, Predictors

## Abstract

**Objectives:**

To identify predictors in patient profiles and to develop, internally validate, and calibrate a screening model for diabetes mellitus (DM) in patients with periodontitis in dental settings

**Materials and methods:**

The study included 204 adult patients with periodontitis. Patients’ socio-demographic characteristics, general health status, and periodontal status were recorded as potential predictors. The diabetic status was considered the outcome, classified into no DM, prediabetes (pre-DM), or DM. Multinomial logistic regression analysis was used to develop the model. The performance and clinical values of the model were determined.

**Results:**

Seventeen percent and 47% of patients were diagnosed with DM and pre-DM, respectively. Patients’ age, BMI, European background, cholesterol levels, previous periodontal treatment, percentage of the number of teeth with mobility, and with gingival recession were significantly associated with the diabetic status of the patients. The model showed a reasonable calibration and moderate to good discrimination with area under the curve (AUC) values of 0.67 to 0.80. The added predictive values for ruling in the risk of DM and pre-DM were 0.42 and 0.11, respectively, and those for ruling it out were 0.05 and 0.17, respectively.

**Conclusions:**

Predictors in patient profiles for screening of DM and pre-DM in patients with periodontitis were identified. The calibration, discrimination, and clinical values of the model were acceptable.

**Clinical relevance:**

The model may well assist clinicians in screening of diabetic status of patients with periodontitis. The model can be used as a reliable screening tool for DM and pre-DM in patients with periodontitis in dental settings.

**Electronic supplementary material:**

The online version of this article (10.1007/s00784-020-03281-w) contains supplementary material, which is available to authorized users.

## Introduction

Periodontitis is a common chronic inflammatory disease that is characterized by the destruction of the supporting structures of the teeth [1]. Based on the Global Burden of Disease (GBD) study 2010, chronic periodontitis is the second most common disease in dentistry with a prevalence of 10.8% in the global population [2]. Diabetes mellitus (DM) is a heterogeneous group of physiological disorders characterized by hyperglycemia resulting directly from insulin resistance, inadequate insulin secretion, or excessive glucagon secretion [3]. It is reported that there were 382 million people worldwide that have DM in 2013, and the number of people with DM by 2035 is expected to rise to 592 million [4].

There is a strong association between periodontitis and DM. DM has been unequivocally confirmed as a major risk factor for periodontitis [5–8]. It is reported that DM can (i) directly change the subgingival periodontal flora due to the increased glucose level in crevicular fluid and blood, (ii) exaggerate the inflammatory host response by producing more inflammatory mediators and inflammatory cytokines in gingival crevicular fluid and gingival tissue, (iii) impair production of bone matrix component, (iv) change the permeability of gingival capillaries, and (v) impair wound healing [8, 9]. Therefore, the reduction in defense mechanisms and the increased susceptibility to infection in patients with DM severely exacerbate the onset, progression, and severity of periodontitis [9]. Based on a meta-analysis including 49,262 individuals, DM increased the risk of incidence or progression of periodontitis by 86% [10]. However, the association between periodontitis and prediabetes (pre-DM) is controversial [11–14].

The consensus has been reached among all the key stakeholders relating to dental teams, including patients, dentists, dental hygienists, dental students, and physicians that the engagement of the dental workforce to identify DM or pre-DM was beneficial [15]. Early screening of DM or pre-DM for patients with periodontitis may guide clinicians to develop different dental treatment strategies for the patients, such as the adjunctive systemic antibiotic use in scaling, root planing, or periodontal surgery [16, 17]. Also, early screening of DM or pre-DM may help reduce patients’ comorbidities and mortality, thus improving patients’ health outcomes [18]. There is growing evidence showing that periodontal treatment leads to an improvement of glycemic control of patients with DM and pre-DM [19–21]. A screening of DM or pre-DM is also an important reminder that the patients need to start with systematic and comprehensive periodontal check-ups or treatment. While DM may remain undetected for some period, around 1 in every 3 people who have DM are not aware of their status [22, 23]. Besides, the prevalence of undiagnosed pre-DM in Korean young adults aged < 40 years was reported to be up to 25% [24]. Therefore, in the decision-making on treatment strategies, it is highly advisable for dentists to verify whether patients with periodontitis suffer from DM or pre-DM at the chairside.

While the use of the glucose meter and finger stick testing has been recommended for dental practices, only a few dental offices own and use a glucose meter [25]. This may be because dentists consider glucose testing out of scope for their daily practice [22, 25], and these recommended tests are invasive. Therefore, the development of a clinical screening model for DM and pre-DM based on patients’ profiles might be of great clinical value in daily practice. Such a clinical screening model should be easy-to-use and acceptable for the clinicians and patients with periodontitis in daily practice.

Therefore, the aims of the present study were to (1) identify potential predictors in patient profiles that allow accurate screening of DM and pre-DM; and (2) develop, internally validate, and calibrate a screening model for DM and pre-DM in patients with periodontitis in dental settings.

## Materials and methods

### Participants

The study was designed as a cross-sectional study. The study involved 204 consecutive patients with periodontitis who were referred to the Department of Periodontology of the Academic Centre for Dentistry Amsterdam (ACTA) for the diagnosis and treatment of periodontitis between February 2014 and September 2015, as previously described by Teeuw et al. 2017 [22]. Patients referred to ACTA for their periodontitis were recruited during their first visit to the periodontal clinic. The inclusion criteria for the patients were: (1) patients were diagnosed with periodontitis based on the Centers for Disease Control and Prevention-American Academy of Periodontology (CDC-AAP) case definition; (2) patients were over 18 years age; and (3) patients provided their informed consent.

The study has been approved by the Medical Ethics Review Committee of the VU University Medical Center Amsterdam (VUmc) (2013.343) and has been performed in accordance with the ethical standards as laid down in the 1964 Declaration of Helsinki and its later amendments or comparable ethical standards.

### Potential predictors

Potential predictors included patient socio-demographic characteristics (age, gender, highest completed education level, and European background), self-reported lifestyle and general health status (smoking, hypertension, hypercholesterolemia, family diabetes, body mass index (BMI)), and periodontal health status (severity of periodontitis, number of teeth, percentage of the number of teeth with ≥ 50% bone loss, percentage of the number of teeth with probing pocket depth (PPD) ≥6 mm, percentage of the number of teeth with mobility, percentage of the number of teeth with gingival recession, bleeding index, and previous periodontal treatment). These potential predictors were identified a priori based on the previous literature and periodontal experts’ knowledge and experience. The details on the definition and measurement of the predictors are shown in Online Resource 1.

### Outcome

The outcome of the study was diabetic status, which was assessed with HbA1c values based on finger-stick test. The details for the finger-stick procedures adopted in the study were presented in Teeuw et al. 2017 [22]. DM status was classified into three categories including “no DM,” “pre-DM,” and “DM” based on the American Diabetes Association (ADA) guidelines [26]. No DM was defined as the HbA1c values < 39 mmol/mol. Pre-DM was defined as the HbA1c values of 39–47 mmol/mol. DM was defined as the HbA1c values ≥ 48 mmol/mol.

### Statistical analysis

#### Screening of potential predictors and modeling

First, collinearity tests of the potential predictors were performed with Spearman rank correlation tests. If the correlation coefficients between two predictors were larger than 0.9, one of the two predictors was excluded from the modeling procedures. The bivariate association of each predictor with the three-category outcome (no DM, pre-DM, and DM) was tested by using the chi-square test for categorical predictors and Kruskal-Wallis test for continuous predictors. Predictors with a bivariate *P* value ≤ 0.25 were selected for inclusion in the subsequent modeling procedure. Multivariable multinomial logistic regression analysis with backward-selection procedures (*P* > 0.25 for removal) was then used for modeling. A less stringent threshold of 0.25 was used in both the bivariate tests, and the multivariable regression analyses in selection and exclusion of potential predictors because this could avoid false negative findings in both modeling stages and avoid unjustified exclusion of predictors from the final model, especially when the sample size is small [27].

#### Shrinkage factor

To improve the internal validity of the model, the regression coefficients of the predictors in the model were multiplied by a shrinkage factor. A shrinkage factor ranges from 0 to 1. The shrinkage factor of the model was calculated as (modelX^2^-*df*)/modelX^2^, where modelX^2^ indicates the likelihood ratio of the fitted model, and *df* indicates the degrees of freedom of the number of candidate predictors considered for the model [28, 29].

#### Calibration

Calibration is defined as the agreement between the predicted outcomes and observed outcomes [30]. Calibration of the model was assessed by plotting the predicted individual probabilities against the observed actual probabilities for each outcome category and by the Pearson goodness-of-fit statistic. If the *P* value of the Pearson goodness-of-fit statistic test is > 0.05, it indicates no or low evidence for lack of fit of the model [31].

#### Discrimination

Discrimination is defined as the ability of a model to differentiate between those with and those without the outcome event [30]. Discrimination of the model was assessed with polytomous discrimination index (PDI) [32] and area under the receiver operating characteristic curve (AUC) [28]. The PDI, which ranges from 0 to 1, is interpreted as the probability of a multinomial model to correctly identify a case from a randomly selected category within a set of K cases (K is the number of categories of a multinomial outcome) [32]. Discrimination of the model was also assessed with two AUCs, relating no DM to the other two outcome categories (DM and pre-DM) in each receiver operating characteristic curve (ROC) area [28].

#### Clinical values

Clinical added values of the model were assessed using the prevalence (prior probability) and posterior probabilities of the outcome categories. The posterior probability was defined as positive predictive values (PPVs) and negative predictive values (NPVs). PPV was defined as the proportions of presence of pre-DM or DM based on the model in patients with pre-DM or DM. NPV was defined as the proportions of absence of pre-DM or DM based on the model in patients with no pre-DM or DM. The PPVs and the prevalence of pre-DM and DM were used to assess the added value of the model for ruling in the risk of pre-DM and DM, while the NPVs and the complement of the prevalence of pre-DM and DM were used to assess the added values of the model for ruling out the risk of pre-DM and DM.

#### Scoring system

A clinical prediction rule for the diabetic status of the patients was developed to provide an estimate for individual patients of their absolute risk of having no DM, pre-DM, and DM. No DM was regarded as the reference category of the outcome, so the predicted probabilities of pre-DM and DM in individual patients were calculated as below [28]:

$$ {P}_{\mathrm{preDM}}=\frac{\exp \left(\mathrm{LPpreDM}\right)}{1+\exp \left(\mathrm{LPpreDM}\right)+\exp \left(\mathrm{LPDM}\right)}; $$

$$ {P}_{\mathrm{DM}}=\frac{\exp \left(\mathrm{LPDM}\right)}{1+\exp \left(\mathrm{LPpreDM}\right)+\exp \left(\mathrm{LPDM}\right)}. $$

where LPpreDM = linear predictor of pre-DM = β0_preDM_ + β1_preDM_X_1_ + … + βi_preDM_*X*_i_; and LPDM = linear predictor of DM = β0_DM_ + β1_DM_X_1_ + … + βi_DM_*X*_*i*_. *β* represents the regression coefficient of a predictor in the model. The status of a patient for any binary variable can be expressed as either 0 or 1, while the status of a patient for any continuous variable can be expressed as its numeric value. As the sum of the predicted probabilities of each outcome category in the model is 1, the probability of patients with no DM can be calculated as 1−P_preDM_−P_DM_. Patients were allocated to the outcome category with the highest predicted probability.

To facilitate the calculation of the probabilities of no DM, pre-DM, and DM in individual patients separately, the multinomial regression model was converted to a score chart. The score of each included predictor in the score chart was produced by the shrunken regression coefficients being divided by the smallest regression coefficient of the predictors and subsequently rounded. Line charts were then developed to help determine the predicted probability of no DM, pre-DM, and DM.

All the statistical procedures mentioned above were performed with SPSS software 25.0 (IBM, New York, the USA) and R software 3.2.3 (R Development Core Team, Vienna, Austria). The discrimination, calibration, added values, and scoring system of the model were all assessed based on the shrunken regression coefficients. Complete-case analysis was used for the missing data of the study.

## Results

A total of 204 patients with periodontitis (98 males and 106 females) were enrolled in the study. Their diabetic status was unknown at study inclusion. The mean age ± standard deviation (SD) of the patients was 50.9 ± 10.9 years. The mean age ± SD of male patients was 50.9 ± 10.5 years while that of female patients was 50.8 ± 11.4 years. Based on their HbA1c values, 35 patients (17%) were classified with DM and 95 patients (47%) with pre-DM.

The correlation coefficients between any two of the potential predictors were smaller than 0.9. The distribution of potential predictors based on the three-category outcome is presented in Table 1**.** A total of 13 predictors had a *P* value of ≤ 0.25 and were selected for possible inclusion in the multivariate multinomial logistic regression analyses using the backward-selection procedure.

**Table 1:** Distribution of the potential predictors based on the diabetic status of patients with periodontitis (*N* = 204)

Predictors	Description of coding	Values	No DM	Pre-DM	DM	*P* value^g^
Socio-demographic characteristics						
Age	Continuous	50.9 ± 10.9 (*N* = 204)	47.6 ± 11.7 (*N* = 74)	53.6 ± 9.6 (*N* = 95)	50.4 ± 11.2 (*N* = 35)	< 0.01
Gender	Male Female	98 106	34 40	45 50	19 16	0.71
Highest completed education level	Low Medium High	49 74 81	14 27 33	20 36 39	15 11 9	0.07
European background	European Non-European	147 57	60 14	69 26	18 17	< 0.01
Self-reported general health status						
Smoking	No Yes	134 70	47 27	60 35	27 8	0.29
Hypertension^a^	No Yes	166 38	65 9	76 19	25 10	0.11
Hypercholesterolemia^a^	No Yes	165 39	67 7	73 22	25 10	0.02
Family diabetes^a^	No Yes	105 99	42 32	47 48	16 19	0.49
BMI^b^	Continuous	26.5 ± 4.5 (*N* = 203)	25.0 ± 4.1 (*N* = 73)	26.4 ± 4.0(*N* = 95)	29.8 ± 4.9 (*N* = 35)	< 0.01
Periodontal health status						
Severity of periodontitis	Mild/Moderate Severe	126 78	51 23	58 37	17 18	0.12
Number of teeth	Continuous	26.0 ± 3.6 (*N* = 204)	26.6 ± 3.1 (*N* = 74)	25.8 ± 3.6 (*N* = 95)	25.4 ± 4.5 (*N* = 35)	0.18
Percentage of the number of teeth with ≥ 50% bone loss (%)^c^	Continuous	16.3 ± 18.1 (*N* = 194)	12.5 ± 13.9 (*N* = 70)	17.4 ± 19.9 (*N* = 91)	21.3 ± 19.4 (*N* = 33)	0.05
Percentage of the number of teeth with PPD ≥ 6 mm (%)	Continuous	36.4 ± 29.7 (*N* = 204)	33.9 ± 29.5 (*N* = 74)	37.5 ± 29.6 (*N* = 95)	38.5 ± 30.4 (*N* = 35)	0.66
Percentage of the number of teeth with mobility (%)^d^	Continuous	14.9 ± 19.3 (*N* = 202)	11.1 ± 14.7 (*N* = 72)	15.5 ± 19.7 (*N* = 95)	21.2 ± 24.8 (*N* = 35)	0.04
Percentage of the number of teeth with gingival recession (%)^e^	Continuous	76.1 ± 25.5 (*N* = 202)	71.5 ± 25.9 (*N* = 72)	77.1 ± 26.7 (*N* = 95)	82.9 ± 19.6 (*N* = 35)	0.09
Bleeding index^f^	Continuous	59.7 ± 29.0 (*N* = 202)	57.1 ± 28.7 (*N* = 72)	57.6 ± 28.8 (*N* = 95)	70.9 ± 28.1 (*N* = 35)	0.04
Previous periodontal treatment^a^	No Yes	109 95	33 41	51 44	25 10	0.03

^a^If a patient’s answer to the question is ‘do not know,’ it is regarded as ‘no’ in the coding; ^b^the data of BMI from one patient was missing; ^c^the data of percentage of the number of teeth with ≥ 50% bone loss from 10 patients were missing; ^d^the data of percentage of the number of teeth with mobility from 2 patients were missing; ^e^the data of percentage of the number of teeth with gingival recession from 2 patients were missing; ^f^the data of bleeding index from 2 patients were missing; ^g^the *P* values were produced from chi-square test for categorical predictors or from Kruskal-Wallis tests for continuous predictors; *DM*, diabetes mellitus; *pre*-*DM*, prediabetes; *BMI*, body mass index; *PPD*, probing pocket depth

In the multivariate modeling, 201 patients were included, while three patients were excluded because of a missing data of one or more predictors in the multivariable model. The predictors included in the final model based on the multivariate multinomial logistic regression analysis are presented in Table 2. When patients with no DM were regarded as the reference, patients with older age, higher BMI, the absence of previous periodontal treatment, non-European background, and presence of hypercholesterolemia were more likely to have pre-DM, while patients with higher BMI, higher percentage of teeth with mobility, higher percentage of teeth with gingival recession, the absence of previous periodontal treatment, non-European background, and presence of hypercholesterolemia were more likely to have DM.

**Table 2:** Multivariate multinomial logistic regression analyses (*P* ≤ 0.25 after backward selection) based on the diabetic status of patients with periodontitis, when no DM was regarded as the reference outcome category (*N* = 201)

	Pre-DM				DM			
Predictors	*β* (SE)	Shrunken *β*	OR (95%CI)	*P* value	*β* (SE)	Shrunken *β*	OR (95%CI)	*P* value
Intercept	− 4.583 (1.387)	− 3.574		0.001	− 10.852 (2.167)	− 8.465		<0.001
Age	0.056 (0.018)	0.044	1.058 (1.021 1.096)	0.002	0.020 (0.026)	0.016	1.020 (0.970 1.073)	0.442
European background								
European	Reference				Reference			
Non-European	0.654 (0.424)	0.509	1.923 (0.838 4.416)	0.123	1.222 (0.545)	0.952	3.393 (1.165 9.881)	0.025
Hypercholesterolemia								
No	Reference				Reference			
Yes	0.627 (0.489)	0.488	1.872 (0.718 4.880)	0.200	0.993 (0.624)	0.774	2.699 (0.795 9.163)	0.111
BMI	0.056 (0.042)	0.044	1.058 (0.974 1.149)	0.183	0.239 (0.057)	0.186	1.270 (1.137 1.419)	< 0.001
Percentage of the number of teeth with mobility (%)	0.011 (0.010)	0.009	1.011 (0.991 1.032)	0.287	0.021 (0.012)	0.016	1.021 (0.997 1.047)	0.091
Percentage of the number of teeth with gingival recession (%)	0.000 (0.007)	0.000	1.000 (0.986 1.014)	0.967	0.014 (0.011)	0.011	1.015 (0.993 1.036)	0.180
Previous periodontal treatment								
Yes	Reference				Reference			
No	0.437 (0.344)	0.340	1.548 (0.789 3.040)	0.204	0.998 (0.510)	0.777	2.712 (0.998 7.369)	0.050

*β*, coefficient; *SE*, standard error; *OR*, odds ratio; *CI*, confidence interval; *DM*, diabetes mellitus; *pre*-*DM*, prediabetes; *BMI*, body mass index

**Fig. 1:**
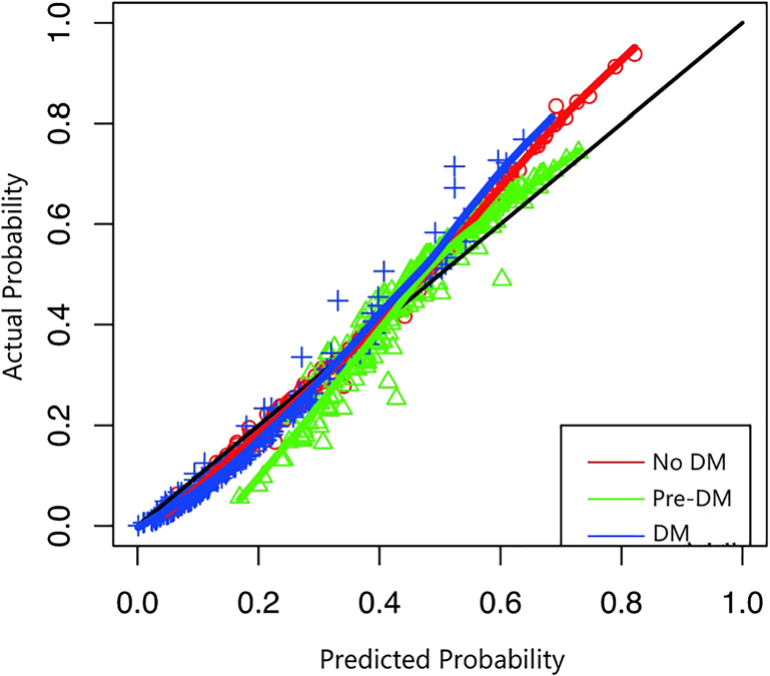
Calibration plots of the multinomial regression model for predicted and actual probabilities of the three outcome categories in patients with periodontitis. The diagonal line is what would result if the predicted probability of the model was the same as the actual probability of the model so that the prediction is neither underestimated nor overestimated. The red curve is the calibration curve for no DM (number of patients with actual no DM is 71, while that with predicted no DM is 58). The green curve is the calibration curve for pre-DM (number of patients with actual pre-DM is 95, while that with predicted pre-DM is 121). The blue curve is the calibration curve for DM (number of patients with actual DM is 35, while number of patients with predicted DM is 22)

**Fig. 2:**
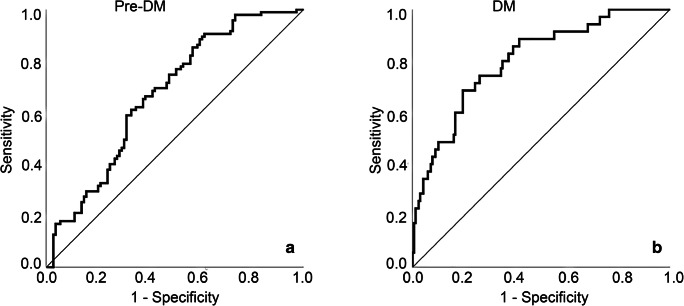
Discrimination ability of the multinomial regression model for screening of DM and pre-DM in patients with periodontitis. **a** is the ROC areas of pre-DM vs no DM and DM with an AUC of 0.67 (95%CI, 0.60, 0.75) and **b** is the ROC areas of DM vs no DM and pre-DM with an AUC of 0.80 (95%CI, 0.72, 0.88)

The shrinkage factor was 0.78. Figure 1 shows the calibration plot of the model. The three curves in the model were all lying close to the diagonal line, which indicated that there was a good fit between the predicted probability and actual probability of the three types of diabetic status of patients with periodontitis. With a resulting *P* value for the Pearson goodness-of-fit test of 0.32, the model was also shown to be a good fit. The PDI of the multinomial model was 0.61. The AUC values for pre-DM and DM were 0.67 (95%CI, 0.60, 0.75) and 0.80 (95%CI, 0.72, 0.88) (Fig. 2). It showed that the general discrimination of the model was moderate to good.

Table 3 presents the clinical values of the model, in aspects of prevalence, sensitivity, specificity, PPV, and NPV. When DM was considered the predicted outcome category, the added value of the model for ruling in the risk of DM was 0.42 (95%CI, 0.20, 0.63) in addition to the prevalence, while that for ruling out the risk of DM was 0.05 (95%CI, − 0.02 0.12) in addition to the compliment of the prevalence. When pre-DM was considered the predicted outcome category, the added value of the model for ruling in the risk of pre-DM was 0.11 (95%CI, 0.00, 0.22) in addition to the prevalence, while that for ruling out the risk of pre-DM was 0.17 (95%CI, 0.05, 0.29) in addition to the compliment of prevalence.

**Table 3:** Clinical values of the model (*N* = 201)

Outcome category	Prevalence (95% CI)	Sensitivity (95% CI)	Specificity (95% CI)	PPV (95% CI)	NPV (95% CI)	Added value for ruling in the risk of (pre)DM (95% CI)	Added value for ruling out the risk of (pre)DM (95% CI)
DM	0.17 (0.13 0.23)	0.37 (0.22 0.54)	0.95 (0.90 0.97)	0.59 (0.38 0.78)	0.88 (0.82 0.92)	0.42 (0.20 0.63)	0.05 (− 0.02 0.12)
Pre-DM	0.47 (0.40 0.54)	0.75 (0.65 0.83)	0.53 (0.43 0.62)	0.59 (0.50 0.67)	0.70 (0.59 0.79)	0.11 (0.00 0.22)	0.17 (0.05 0.29)

*CI*, confidence interval; *PPV*, positive predictive value; *NPV*, negative predictive value; *DM*, diabetes mellitus; *pre-DM*, prediabetes

**Fig. 3:**
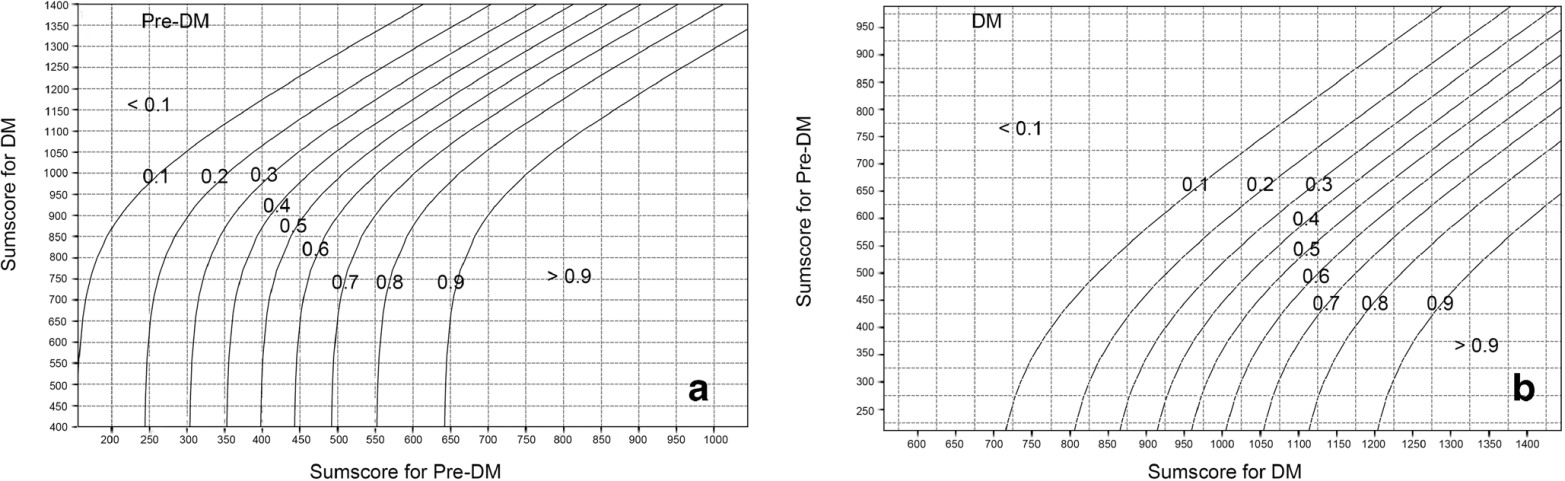
Line charts of the multinomial regression model for determining the predicted probability of **a** pre-DM and **b** DM. The cross point of a vertical line drawn from the x-axis and a horizontal line drawn from the y-axis shows the corresponding predicted probability of the outcome category. The corresponding predicted probability of no DM can be calculated by 100% minus the predicted probability of pre-DM minus the predicted probability of DM

To enhance the clinical usefulness of the model, the final multinomial regression model was transformed into a score chart based on the shrunken regression coefficients (Table 4). A clinician can easily calculate the sum scores for pre-DM and DM of individual patients separately based on the predictors in the score chart. Then, a clinician can determine the corresponding predicted probability of pre-DM and DM based on the sum scores for pre-DM and DM by using the line charts (Fig. 3). The predicted probability of no DM can be calculated by 1 minus the predicted probability of pre-DM minus the predicted probability of DM.

**Table 4:** Score chart of the multinomial model for prediction of diabetic status of patients with periodontitis (*N* = 201)

	Pre-DM	DM
Predictors	Score	Score
Age	5*age	2*age
European background		
European	0	0
Non-European	57	106
Hypercholesterolemia		
No	0	0
Yes	54	86
BMI	5*BMI	21*BMI
Percentage of the number of teeth with mobility (%)	100*%	200*%
Percentage of the number of teeth with gingival recession (%)	0*%	100*%
Previous periodontal treatment		
Yes	0	0
No	38	86
Sum score		

*DM*, diabetes mellitus; *pre*-*DM*, prediabetes; *BMI*, body mass index

The algorithms for the calculation of an individual’s sum scores for pre-DM and DM were emerged from the modeling:

*Sum score for pre-DM* = 5*Age + 57*non-European + 54*presence of hypercholesterolemia + 5*BMI + 100*percentage of the number of teeth with mobility + 38*no previous periodontal treatment

Sum score for DM = 2*age + 106*non-European + 86*presence of hypercholesterolemia + 21*BMI + 200*percentage of the number of teeth with mobility + 100*percentage of the number of teeth with gingival recession + 86*no previous periodontal treatment

For example, a patient with periodontitis came to the clinic to seek treatment. He was a European and 50 years old. His self-reported BMI was 25. He had hypercholesterolemia and received no previous periodontal treatment. He had a total of 24 remaining teeth, while 12 teeth were mobile, and 20 teeth had gingival recession. Therefore, based on the score chart (Table 4), the patient had a sum score for pre-DM of 517 and a sum score for DM of 980. Then, based on the line charts (Fig. 3), the predicted probability for pre-DM can be estimated to be around 52% and that for DM around 28%. The predicted probability for no DM can be calculated as 1*−*52%−28% = 20%. Therefore, the patient was very likely to have a pre-DM because the predicted probability for pre-DM was the highest.

## Discussion

Based on the common and easily obtainable clinical variables, the present study derived a model for the screening of the diabetic status of patients with periodontitis. To the authors’ knowledge, this is the first screening model for the diabetic status in patients with periodontitis in particular. There are already existing models for identification of DM in dental offices for the general dental patients [33–35], and for the prediction of the incident DM in the future in medical patients and general population [36–39]. In these previous models, patients’ age, ethnicity, BMI, waist circumference, family history of DM, hypertension, and some biomarkers such as lipids level, uric acid level, and *γ*-glutamyltransferase level were the important predictors for DM. Some of these predictors, like age, European background, and BMI were also included in the present model. However, patients’ self-reported family history of DM and hypertension were excluded from the present model, while several new periodontal related predictors, like percentage of the number of teeth with mobility and percentage of the number of teeth with gingival recession, were included in the present model. This may be because the target population of the present model is patients with periodontitis, so the effects of periodontal-related predictors on the outcome might be larger than other predictors, thus taking over the places in the model. In the present model, the biomarkers were not considered candidate predictors because laboratory tests are not routinely adopted for patients in dental clinics. Furthermore, the previous models targeted general population, medical patients, or general dental patients, but the present model particularly targeted patients with periodontitis. This is because much evidence has shown the strong bidirectional associations between DM and periodontal diseases. It is reasonable and necessary that the dental offices can be an important health-care location actively involved in screening for unidentified DM of periodontal patients. Besides, very few previous models have identified pre-DM separately from DM in dental settings. Pre-DM is a critical risk state that defines a high chance of developing diabetes. It is reported that for pre-diabetic individuals, lifestyle modification is the cornerstone of DM prevention with evidence of a 40–70% relative risk reduction [40]. Early screening of pre-DM may prevent patients’ diabetic status from being more severe and irreversible. So, early screening of pre-DM is as important as that of DM in clinical practice. That is why pre-DM and DM were treated as two separately outcome categories in the present model.

The risk of diabetes can be also identified by the National Institute for Health and Clinical Excellence (NICE) guidance tool in dental settings, based on patients’ age, gender, ethnicity, family history of diabetes, hypertension, BMI, and the blood test at baseline [41]. The NICE guidance aimed to prevent or delay the progression of pre-DM and DM among high-risk groups, while the prediction model of the study aimed to identify the patients who have DM or pre-DM currently [42]. Therefore, the purposes of using these two screening tools are different. Besides, the NICE guidance was developed by the stakeholders’ consensus, while the prediction model of the study was developed based on the mathematic approach. The individual predicted probability of getting DM cannot be calculated from the NICE guidance tool, and the predictive performance of the tool may not be quantitatively assessed.

The present model provides clinicians with information on patients’ potential diabetic status. For periodontal clinicians, it is important to know the diabetic status of the patients with periodontitis. This is because, on one hand, the incidence and severity of periodontitis are influenced by the presence or absence of DM, and the degree to which DM is controlled by patients. On the other hand, if periodontal clinicians know that the patients have DM or pre-DM, they can refer the patients to physicians or give patients advice on the modification of lifestyles in time. For patients, it is also important to realize their own diabetic status so that patients can seek physicians’ help in time. In clinical practice, the glycohemoglobin (HbA1c) test is considered an appropriate alternative to fasting plasma glucose for early screening of DM and pre-DM [43]. However, such test is bothersome for dental patients because it is invasive, time-consuming, expensive, and less accessible in dental settings. Therefore, the development of the present model can make the screening of DM and pre-DM in dental settings more user-friendly by making it non-invasive, faster, cheaper, and accessible for every patient. This can help in reducing patients’ burden for the diagnosis of DM to a large extent.

Both the discrimination and calibration of the model were acceptable in general, which indicated that the performance of the model may be suitable in clinical practice. However, clinicians need to be aware that a false positive prediction can lead to unnecessarily extended examinations or treatments for DM or pre-DM in patients with periodontitis, thereby adding to the financial and psychological burdens. Similarly, a false negative prediction can lead to undertreatment for diabetic or prediabetic conditions, which may result in less desirable health outcomes. Based on the present model, the risk of a false positive prediction of DM and pre-DM was 0.04 and 0.25, respectively, while the risk of a false negative prediction of DM and pre-DM was 0.11 and 0.12, respectively. This indicates a high risk of false positive prediction of pre-DM, thereby suggesting that 25% of the patients with periodontitis may have a false positive prediction of pre-DM. However, the negative consequence of the false positives for the patients does not seem to be very severe. In addition, this model can be used as a chairside screening tool, allowing further confirmation of the (pre)DM based on physicians’ diagnosis. Therefore, false positive prediction is not likely to affect patients’ health outcomes, indicating the risk of false positives and false negatives as acceptable by this model.

In the present study, the added predictive values of the model for ruling in DM and pre-DM were 0.42 and 0.11, respectively, while those for ruling it out were 0.05 and 0.17, respectively. That indicates that if a patient with periodontitis is predicted to have DM or pre-DM based on the model, the posterior risk of having DM or pre-DM of this patient can be increased by 0.42 and 0.11 compared with the prevalences of DM and pre-DM in patients with periodontitis. Similarly, if a patient with periodontitis is predicted not to have DM (i.e., the patient is predicted to have “no DM” or “pre-DM”) or pre-DM (i.e., the patient is predicted to have “no DM” or “DM”) based on the model, the posterior risk of not having DM or pre-DM of this patient can be increased by 0.05 and 0.17, respectively, when compared with the complement of the prevalences of DM or pre-DM in patients with periodontitis. It should be noted that the added value of the model for ruling out DM is only 0.05, which seems to add only little benefit. However, the prevalence of DM in patients with periodontitis is only 17%. This means that the majority (83%) of the patients do not have DM. Therefore, there is only little space for the model to add more value to the complement of the prevalence of DM. In this case, 5% of the added value can be acceptable. Therefore, the added values of the model for ruling in and out DM and pre-DM are considerable, so it adds to accurate prediction of DM and pre-DM.

Sample size is typically a severe problem for multinomial regression models because one or more of the outcome categories often have very low prevalence [32]. For the multinomial model with three outcome categories, the events per variable (EPV) are advised to be larger than 20 [28]. Insufficient sample size may underestimate the importance of the potential predictors and tends to make the clinically important predictors insignificant. Therefore, insufficient sample size may negatively impact the robustness of the performance of the model [44]. However, the present study did not meet the criterion because of the small sample size, which was a limitation of the study. That is why, in the present study, a less stringent threshold of *P* value of 0.25 was used in both the bivariate tests and multivariate regression analyses in the selection and exclusion of potential predictors. This could avoid false negative findings in both modeling stages to a large extent, especially when the sample size is small [27+]. Another limitation of the study is that the discrimination of the model in terms of pre-DM is not satisfactory based on the current predictors. This may be because pre-DM is an intermediate status between DM and no DM, and the distinction between pre-DM and DM and between pre-DM and no DM was not as large as that between DM and no DM. So, it is more difficult for the model to discriminate the patients with pre-DM from the patients with DM or with no DM. Therefore, researchers are suggested to look for and test other predictors in the future, which are relevant to the occurrence and progression of pre-DM, to update and improve the performance of the current model. Another limitation is the clinical feasibility of some predictors of the model. For example, complete measurements of tooth mobility and full mouth gingiva recession may not be routinely collected in every dental office. The model may add extra workload to clinicians to collect those predictors for each individual patient. Besides, hypercholesterolemia and BMI in the model are patients self-reported. The validity of these self-reported predictors may be questionable. For example, hypercholesterolemia may be underestimated by patients’ self-reporting [45], and this may bias the predictive performance of the model.

Future researchers are suggested to externally validate the present model in other populations and to assess the added values of other relevantly important predictors such as waist circumference and biomarkers including lipids level, uric acid level, and *γ*-glutamyltransferase level to the performance of the model. Besides, it is recommended to explore more dental predictors for DM and pre-DM as they can be more easily collected by dentists at the chairside.

## Conclusions

Potential predictors in patient profiles for screening of DM and pre-DM in patients with periodontitis were identified. The multinomial regression model for screening of DM and pre-DM was developed and internally validated. The calibration and discrimination of the model were acceptable. The added predictive values were considerable for both ruling in and ruling out DM and pre-DM in decision-making. The model can be used as a reliable screening tool for DM and pre-DM in patients with periodontitis in dental settings.

## Electronic supplementary material

ESM 1 (DOCX 14 kb)
