# Supplementary material for: Development and validation of a screening model for diabetes mellitus in patients with periodontitis in dental settings
Source: Clin Oral Investig. 2020 Jun 15;24(11):4089–100. doi: 10.1007/s00784-020-03281-w (PMC7544748; doi:10.1007/s00784-020-03281-w)
Supplement: Supplementary file 1 — (DOCX 14 kb) [file 784_2020_3281_MOESM1_ESM.docx]

**Online Resource 1** Measurement and definition of the potential predictors

| Predictors | Measurement and definition |
| --- | --- |
| **Socio-demographic characteristics** | |
| Age | Self-reported |
| Gender | Self-reported |
| Highest completed education level | Self-reported based on ISCED.^1^ Low education refers to primary education, lower vocation education, and lower secondary education. Medium education refers to intermediate vocational education, and higher secondary education. High education refers to higher vocational education and university. |
| European background | Self-reported |
| **Self-reported general health status** | |
| Smoking | Self-reported with a question “Do you smoke?” |
| Hypertension | Self-reported with a question “Have you ever been told by a doctor/nurse that you had high blood pressure?” |
| Hypercholesterolemia | Self-reported with a question “Have you ever been told by a doctor/nurse that you had a high cholesterol level in your blood?” |
| Family diabetes | Self-reported with a question “Does someone from your grandparents, parents, brothers, or sisters suffer from diabetes?” |
| BMI | Self-reported |
| **Periodontal health status** | |
| Severity of periodontitis | Assessed by clinicians based on CDC-AAP case definitions.^2^ Mild periodontitis was defined as two or more interproximal sites with CAL ≥3mm and two or more interproximal sites with PPD ≥ 4mm (not on the same tooth) or one site with PPD ≥ 5 mm. Moderate periodontitis was defined as two or more interproximal sites with CAL ≥ 4mm (not on the same tooth) or two or more interproximal sites with PPD ≥ 5mm (not on the same tooth). Severe periodontitis was defined as two or more interproximal sites with CAL ≥ 6mm (not on the same tooth) and at least one interproximal site with PPD ≥ 5 mm. |
| Number of teeth | Assessed by clinicians |
| Percentage of the number of teeth with ≥50% bone loss (%) | Bone loss assessed with dental radiographs |
| Percentage of the number of teeth with PPD ≥6mm (%) | PPD recorded at 6 sites per tooth (mesial, mid, and distal on both buccal and lingual surfaces) by clinicians. The number of teeth with PPD ≥6 mm was defined as the number of teeth in which at least one site out of the six sites had a PPD ≥6 mm. |
| Percentage of the number of teeth with mobility (%) | Mobility was assessed by clinicians. The number of teeth with mobility was defined as the number of teeth that had at least 1 mm horizontal mobility |
| Percentage of the number of teeth with gingival recession (%) | Gingival recession was recorded at six sites per tooth (mesial, mid, and distal on both buccal and lingual surfaces) by clinicians. The number of teeth with gingival recession was defined as the number of teeth in which at least one out of the six sites had a positive gingival recession. |
| Bleeding index | Bleeding was assessed by clinicians. Bleeding index of the teeth was defined as the total number of bleeding sites after probing divided by the total number of available sites of the present teeth. |
| Previous periodontal treatment | Self-reported with a question “Has there ever been a periodontal cleansing?” |

ISCED, International Standard Classification of Education; CDC-AAP, the Centers for Disease Control and Prevention-the American Academy of Periodontology; PPD, probing pocket depth; CAL, clinical attachment loss.

**Reference**

1. [Eke PI](https://www.ncbi.nlm.nih.gov/pubmed/?term=Eke%20PI%5BAuthor%5D&cauthor=true&cauthor_uid=22420873), [Page RC](https://www.ncbi.nlm.nih.gov/pubmed/?term=Page%20RC%5BAuthor%5D&cauthor=true&cauthor_uid=22420873), [Wei L](https://www.ncbi.nlm.nih.gov/pubmed/?term=Wei%20L%5BAuthor%5D&cauthor=true&cauthor_uid=22420873), [Thornton-Evans G](https://www.ncbi.nlm.nih.gov/pubmed/?term=Thornton-Evans%20G%5BAuthor%5D&cauthor=true&cauthor_uid=22420873), [Genco RJ](https://www.ncbi.nlm.nih.gov/pubmed/?term=Genco%20RJ%5BAuthor%5D&cauthor=true&cauthor_uid=22420873). Update of the case definitions for population-base surveilance of periodontitis. *J Periodontol* 2012; 83: 1449-1454.

2. [Teeuw WJ](https://www.ncbi.nlm.nih.gov/pubmed/?term=Teeuw%20WJ%5BAuthor%5D&cauthor=true&cauthor_uid=28316794), [Kosho MX](https://www.ncbi.nlm.nih.gov/pubmed/?term=Kosho%20MX%5BAuthor%5D&cauthor=true&cauthor_uid=28316794), [Poland DC](https://www.ncbi.nlm.nih.gov/pubmed/?term=Poland%20DC%5BAuthor%5D&cauthor=true&cauthor_uid=28316794), [Gerdes VE](https://www.ncbi.nlm.nih.gov/pubmed/?term=Gerdes%20VE%5BAuthor%5D&cauthor=true&cauthor_uid=28316794), [Loos BG](https://www.ncbi.nlm.nih.gov/pubmed/?term=Loos%20BG%5BAuthor%5D&cauthor=true&cauthor_uid=28316794). Periodontitis as a possible early sign of diabetes mellitus. *BMJ Open Diabetes Res Care* 2017; 5: e000326.
